# The Association of Midday Napping With Hypertension Among Chinese Adults Older Than 45 Years: Cross-sectional Study

**DOI:** 10.2196/38782

**Published:** 2022-11-22

**Authors:** Dongfeng Tang, Yiheng Zhou, Chengxu Long, Shangfeng Tang

**Affiliations:** 1 School of Medicine and Health Management Tongji Medical College Huazhong University of Science and Technology Wuhan China; 2 Institute for Hospital Management Tsinghua University Shenzhen China; 3 College of Medical, Veterinary, and Life Sciences University of Glasgow Glasgow United Kingdom; 4 Department of Global Health and Social Medicine King's College London London United Kingdom; 5 Research Center for Rural Health Service Key Research Institute of Humanities and Social Sciences of Hubei Provincial Department of Education Wuhan China

**Keywords:** hypertension, risk factor, midday napping, BMI, mediation effect

## Abstract

**Background:**

Hypertension is one of the main public health issues around worldwide, and midday napping is a popular habit. The association between the two remains to be explored.

**Objective:**

The goal of the research was to explore the association of midday napping with hypertension.

**Methods:**

This study separately selected 11,439, 12,689, and 9464 Chinese respondents aged over 45 years from the China Health and Retirement Longitudinal Study 2011, 2015, and 2018 data sets. Binary logistic regression was used to explore the association of midday napping with hypertension, and the 3-step method was used to test the mediation effect of BMI.

**Results:**

Among all respondents, the prevalence rates of hypertension were 24.6% (2818/11439) in 2011, 21.1% (2683/12689) in 2015, and 22.1% (2092/9464) in 2018. Midday napping was positively correlated with hypertension. In 2011 and 2015, napping 60 to 90 minutes had the greatest odds ratios [OR] (OR_2011_ 1.705, OR_2015_ 1.494). In 2018, the biggest OR came from the group napping 30 to 60 minutes (OR 1.223), and ORs of different napping durations decreased from 2011 to 2018. In addition, BMI had a partial mediation effect in 2015 and 2018.

**Conclusions:**

Midday napping is a potential risk factor for hypertension with BMI acting as a mediator. To prevent hypertension, avoiding prolonged duration of midday napping and taking action to maintain a normal BMI level are recommended.

## Introduction

Hypertension is one of the main public health issues worldwide, and it has been identified as one of the main risks for stroke, heart failure, and cerebrovascular disease [1-3]. As of 2019, 1.3 billion people, or more than 16% of the world’s population, are living with hypertension [4]. It has been estimated to contribute to 50% of coronary heart disease cases and two-thirds of the cerebrovascular disease burden [5]. Successive population surveys conducted in China over the last 30 years have revealed an increasing prevalence of hypertension [6,7]. Now there are 270 million hypertensive patients in China, and it has become the main culprit for disability-adjusted life years, contributing to 24.6% of all-cause mortality [8,9].

Considering the high prevalence and enormous health toll, a series of actions have been taken in China. In 2009, the New Health Care System Reform was introduced, and hypertension management was made a vital public health service free for all patients [10]. It was stipulated that primary health care facilities must provide residents with free screening, management, and follow-up services [11]. Additionally, the Chinese central government has constructed many national demonstration areas for community-based hypertension management and comprehensive prevention and control of hypertension to improve the lifestyle and health literacy of the population [12]. The turning point came when the Primary Health Care, Medicine, and Health Promotion Law, pioneering legislation for health promotion in China, was implemented in 2020. It established the legality and necessity of a population-wide hypertension prevention and control approach [13]. As a result, the long ignored prevention of hypertension is being addressed, emphasizing the improvement of modifiable risk factors as a public priority.

Previous studies have identified some modifiable risk factors related to hypertension, including excessive drinking, smoking, unhealthy diet, and lack of exercise [14-16]. Some researchers spotted the link between sleep and hypertension and concluded that sleep duration and quality were strongly associated with the risk of hypertension [17-19]. However, the effect of midday napping, another popular sleep activity, has rarely been addressed. Although some studies indicated an independent association between midday napping and the incidence of hypertension [20-22], study results conflicted. Additionally, the association of hypertension with overweight and obesity has been extensively proven, and the prevalence of hypertension among the obese population may range from 60% to 77%, increasing with BMI [23,24]. Prolonged midday napping duration was found to elevate cortisol levels, resulting in abnormal fat distribution [25]. In addition, decreased thermogenesis and energy expenditure and an activated sympathetic nervous system caused by midday napping may also contribute to obesity [26,27]. Therefore, BMI seems an appropriate mediator to explore the association between midday napping and hypertension and help understand the underlying mechanism. Thus, 3 samples (2011, 2015, 2018) from the China Health and Retirement Longitudinal Study were used in this study to examine the relationship between midday napping and hypertension among middle-aged and older Chinese people and test the mediation effect of BMI. By identifying the potential risk modifiable factors, this study aimed to influence individual lifestyles and public policy to control hypertension.

## Methods

### Sample and Data Collection

The primary data used in this study are from the China Health and Retirement Longitudinal Study, a longitudinal national study conducted in 450 neighborhoods and village committees in 150 counties across 28 provinces. A 4-stage, stratified, cluster probability sampling design was adopted in the baseline survey, and detailed sampling procedures were shown in the study by Wang et al [28]. Data regarding individual demographic and socioeconomic status, health conditions, and related behavior information were collected among residents aged 45 years and older in China. Participants were excluded for the following reasons: aged younger than 45 years, values missing for BMI or height and weight, and information on hypertension missing. The final sample sizes are 11,439 in 2011, 12,689 in 2015, and 9464 in 2018.

### Ethics Approval

The study was approved by the institutional review board of Peking University Health Science Center (IRB approval number for the main household survey, including anthropometrics: IRB00001052-11015; IRB approval number for biomarker collection: IRB00001052-11014). All participants provided their written informed consent before completing the interview.

### Variables

#### Primary Dependent Variable

The dependent variable was a binary variable indicating whether a resident suffered from hypertension. Hypertension is defined in accordance with the national guidelines for primary hypertension prevention and management [29,30]: currently taking antihypertensive drugs, previously diagnosed as hypertensive by a clinician, or systolic blood pressure over 140 mm Hg or diastolic blood pressure over 90 mm Hg without antihypertensive drugs.

#### Primary Independent Variable

Midday napping was set as the independent variable, grouped by napping duration, which was appraised using a self-reported questionnaire [31] that asked, “During the past month, how long did you take a nap after lunch on average?” According to existing literature, categories ranging from no napping to napping longer than 90 minutes were defined (see Multimedia Appendix 1 for data) [32,33].

#### Control Variables

Sociodemographic characteristics (gender, age, education, residential status, marital status, household annual income per capita), health-related variables (self-reported health status, activities of daily living [ADL], mental health, personal medical histories, BMI, lifestyles (smoking status, drinking status, and night sleep duration) were included in this study (see Multimedia Appendix 1 for data) [34-38]. The information was collected by using a structured questionnaire. Age and household annual income per capita were set as continuous variables, and household annual income per capita was log transformed [39]. Participants were categorized as ADL impaired if they reported difficulty or inability performing any activity item [40]. Mental health was appraised using the 10-item Center for Epidemiological Studies Depression Scale (<10=no depressive symptoms and ≥=depressive symptoms) [41]. Cardiovascular diseases were self-reported as chronic heart problems, stroke, or both [42]. BMI was categorized as underweight (BMI <18.5), normal (18.5≤BMI<25.0), overweight (25.0≤BMI<30.0), and obese (BMI ≥30) [43].

### Data Analysis

The disparity in hypertension across different groups was examined by chi-square and independent sample *t* test. After adjusting for control variables, binary logistic regression was used to explore the relationship between midday napping and hypertension. Variables in the regression model were selected using the Enter method. The association between midday napping and hypertension was quantified using odds ratios (ORs) having 95% confidence intervals, with other variables controlled. To verify whether BMI played a role in the influence of midday napping on hypertension, the 3-step method proposed by Baron and Kenny [44] was used to test the mediating effect of BMI. The judging criteria for whether there was a mediation effect were taken as follows: statistically significant relationship between independent variable (X, coefficient=a) and mediator (M), significant relationship between independent variable (X, coefficient=c) and dependent variable (Y), and coefficient of mediator (coefficient=b) in the regression model that contained independent variable, mediator, and dependent variable is statistically significant [45]. Mediator was defined as complete if the coefficient of X was not significant in the regression model including X, M, and Y and partial if the coefficient of X was still significant in the regression model, indicating other remaining factors in the path from X to Y. The mediation effect value was calculated as a*b, and the ratio of the mediating effect with the total effect was calculated as a*b/c [46]. *P*<.05 (2-tailed) was regarded as statistically significant. The data were described and analyzed using SPSS (version 24.0, IBM Corp).

## Results

### Sample Characteristics

There was a reasonably steady percentage of participants overall who had hypertension: 24.64% (2818/11,439) in 2011, 21.14% (2683/12,689) in 2015, and 22.10% (2092/9464) in 2018. Participants who regularly took midday naps were 54.19% (6166/11,439) in 2011, 58.39% (7409/12,689) in 2015, and 60.51% (5727/9464) in 2018. Among all midday nappers, those who napped between 60 and 90 minutes were the largest group, with 23.88% (2717/11,439) in 2011, 27.43% (3480/12,689) in 2015, and 23.63% (1717/9464) in 2018. There were slightly more female participants than male (6018 vs 5421 in 2011, 6424 vs 6265 in 2015, and 4971 vs 4493 in 2018), with an average age of 59.5 years in 2011, 61.0 years in 2015, and 60.6 years in 2018. More information can be found in Multimedia Appendix 1.

### Association Between Midday Napping and Hypertension

**Figure 1:**
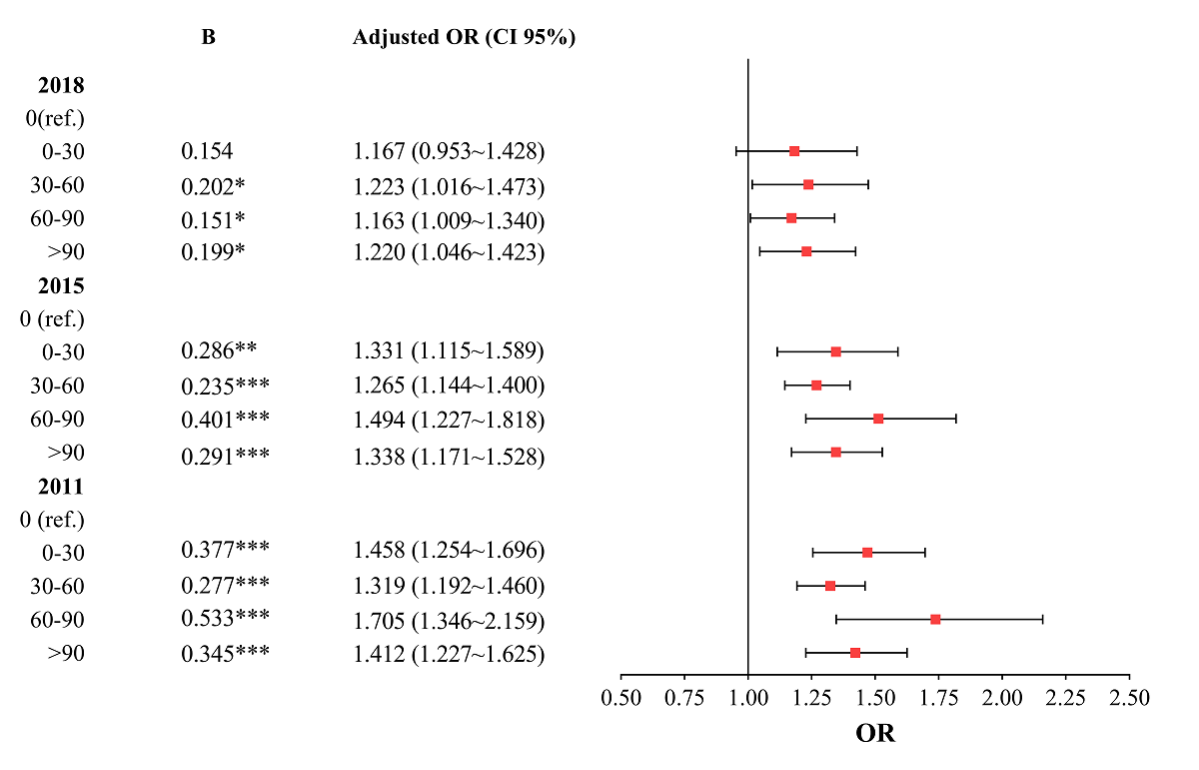
Influence of midday napping on hypertension in different years. OR: odds ratio. *P<.05, **P<.01, ***P<.001. The horizontal line at the end of each line represents the 95% confidence interval, the square in the middle line represents the OR value, and the line segment intersects with the middle vertical line (=1), which means that the result is not significant (*P*>.05). Non-intersection means that the result is significant (*P*<.05). Unit: minute.

Midday napping was found to be positively correlated with hypertension. In 2011 and 2015, after being adjusted by other control variables, groups of nappers (considering the napping durations 0-30, 30-60, 60-90, and >90 minutes) were all positively correlated with hypertension. Napping 60 to 90 minutes had the greatest ORs (2011: OR 1.705, 95% CI 1.346-2.159; 2015: OR 1.494, 95% CI 1.227-1.818) compared with nonnappers. In 2018, except for the group napping 0 to 30 minutes, participants were positively correlated with hypertension, and the greatest OR came from the group napping 30 to 60 minutes (OR 1.223, 95% CI 1.016-1.473). See Figure 1.

**Figure 2:**
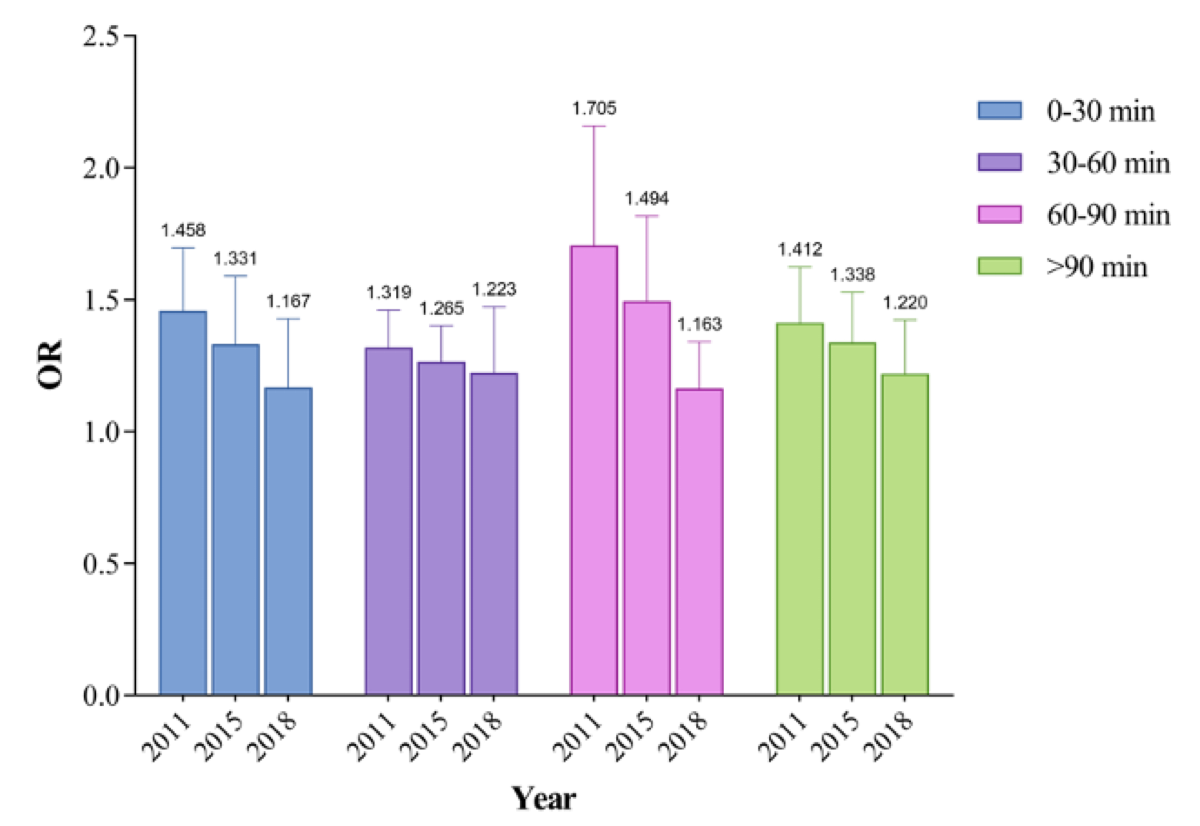
Trajectories of odds ratios from 2011 to 2018. The odds ratio of napping 0 to 30 minutes was not significant in 2018. OR: odds ratio.

From the longitudinal perspective, the ORs of each group of nappers decreased from 2011 to 2018. ORs of napping 60 to 90 minutes decreased the most, from 1.705 in 2011 to 1.338 in 2015 and 1.163 in 2018. The ORs of napping more than 90 minutes decreased from 1.412 in 2011 to 1.220 in 2018. The ORs of napping 30 to 60 minutes decreased from 1.319 in 2011 to 1223 in 2018. Last, the ORs of napping 0 to 30 minutes decreased from 1.458 in 2011 to 1.331 in 2015. See Figure 2.

### Mediation Effect of BMI

The data revealed that the mediation effect of BMI existed in 2015 and 2018 but not in 2011. In 2015, the total effect of midday napping was found to be significant on hypertension (path c: B 0.012, *P*<.001). Midday napping had a positive effect on BMI in path a (B 0.011, *P*<.01), and BMI had a positive effect on hypertension in path b (B 0.022, *P*<.001). In path c', the effect of midday napping was also significant (B 0.012, *P*<.001), so the BMI was identified as a partial mediator. The mediation effect was 0.000242, with a ratio of 2.01% to the total effect. In 2018, the coefficients in path a, path b, and path c were also found to be significant, and the mediation effect of BMI was identified as partial, reaching 0.003058. The ratio of the mediation effect over the total effect increased to 23.52% (see Table 1).

**Table 1:** Coefficient (B) in testing model of mediation effect.

Year	X^a^–Y^b^(path c^c^)	X–M^d^(path a^e^)	(X+M)–Y	
			X(path c'^f^)	M(path b^g^)
2011	0.018^h^	–0.023^h^	0.017^h^	–0.005
2015	0.012^h^	0.011^i^	0.012^h^	0.022^h^
2018	0.013^h^	0.022^h^	0.010^h^	0.139^h^

^a^X: midday napping time.

^b^Y: hypertension.

^c^path c: regression between X and Y.

^d^M: BMI.

^e^path a: regression between X and M.

^f^path c: regression between X and Y with M controlled.

^g^path b: regression between M and Y with X controlled.

^h^*P*<.001.

^i^*P*<.01.

## Discussion

### Principal Findings

This cross-sectional study found midday napping positively associated with hypertension among 3 sectional samples in China, indicating that midday napping may represent a potential causal risk factor. Meanwhile, the ORs of various napping duration decreased over time. The BMI was found to be a partial mediator between midday napping and hypertension.

Although napping has long been regarded as a healthy habit, this study suggests that it may be a potential risk factor for hypertension. Evidence from the UK Biobank [47] and cohort studies in China [33] also supported the results of this study. A meta-analysis concluded that the pooled relative risk of hypertension in nappers was 1.13 based on 9 observational studies [48]. However, disparities between this study and existing literature also exist, indicating the need for a cautious interpretation of the results. Some other studies found midday napping to have a protective effect for habitual nappers compared with those who never napped [49] or to decrease the risk of hypertension in specific napping durations [50,51], contradictory to the results in this study. Meanwhile, in this study, different durations of midday napping were all found to be positively associated with hypertension (except napping for 0 to 30 minutes in 2018). However, the associations of midday napping duration with hypertension differed in various studies. For example, the significantly increased odds for hypertension were only found in participants napping over 90 minutes in some studies [32,35]. Another cohort study conducted in China including 13,706 participants found no significant associations of napping for less than 30 minutes with hypertension [36]. The inconsistency might be explained by study designs and samples, different characteristics of participants, disparity in included confounders, and measurements of napping behaviors and other confounders across studies. Therefore, it is important to be cautious about the results, and long-term follow-up and experimental studies are needed to determine the exact impacts of midday napping.

From 2011 to 2018, decreases in ORs were seen in different napping durations (napping 0-30 minutes: 1.458 to 1.331; napping 30-60 minutes: 1.319 to 1.223; napping 60-90 minutes: 1.705 to 1.338; napping over 90 minutes: 1.412 to 1.220). Some speculations were made to understand the results. First, there were only 4 variables significant (including education, marital status, drinking, and napping duration) in the regression model of 2011, but the corresponding number was 8 in 2018 ( including gender, age, health status, ADL, diabetes, cardiovascular disease, BMI, and napping duration). The increasing correlation between significant variables might decrease the value of ORs. Second, the great socioeconomic and environmental transformations related to hypertension during 2011-2018, such as dietary patterns, exposure to fine particulate matter (PM_2.5_), built environment factors, and some other confounders, were not controlled in our study [52-54]. Third, the association of midday napping with hypertension might be moderated by other variables such as physical conditions and night sleep duration [49,55]. Therefore, current evidence was not enough to conclude that the impact of midday napping decreased, and this can only be explained after determining the hidden specific mechanism. However, the results deserve our attention because they indicate the possibility that the potential risk of midday napping might be mitigated or even eliminated if we can control other confounders well.

The mediation effect of BMI was identified in this study. Previous studies found that nappers were more likely to be overweight or obese [34,56,57], so it could be inferred that midday napping contributes to hypertension by elevating the risk of obesity or overweight, which is an acknowledged risk factor for hypertension [58-60]. However, the ratio of mediation effect over the total effect was 2.01% in 2015 and 23.5% in 2018, indicating the existence of other mediators. It was suggested that midday napping could result in sympathetic surge and an increase in nighttime cortisol, elevating blood pressure [25]. Midday napping was also regarded as a symptom of sleep apnea, and it was concluded that the sleep apnea and not the napping itself resulted in cardiovascular diseases [61]. Furthermore, prolonged midday napping may have an impact on the duration and quality of evening sleep [62]. All these factors can indirectly increase the risk of hypertension.

To prevent hypertension, prolonged midday napping should be avoided, and actions related to losing weight such as increased physical activity and a balanced diet are also needed, especially for nappers. Additionally, further research is needed to define the vulnerable population and develop corresponding interventions.

### Limitations

In this study, there were some limitations that should be mentioned for cautious interpretation. First, despite the positive correlation observed, the regression model and cross-sectional study design used were not robust enough to conduct the causal inference, which weakened the evidence. Second, the use of self-reported midday napping duration and some other variables might introduce recall bias. Third, although some confounders were adjusted in the model, potential residual covariates might remain due to the absence of information such as genetic factors, family history of hypertension, biomarkers, and environmental factors. In addition, time-dependent covariates were not included in our study, which made comparisons across years difficult. Fourth, all participants were aged 45 years and older, and it remains uncertain whether the conclusion can be applied to other age groups. Additionally, although we added night sleep duration as a control variable, the potential interaction effect of midday napping and night sleep was not analyzed in this study.

### Conclusion

In this study, it was found that midday napping was positively associated with hypertension in Chinese people middle-aged and older. Although the causal effects were hard to prove, BMI was found to play the role of mediator. Therefore, avoiding prolonged midday napping and taking action to maintain a normal BMI level are recommended. For future research, the specific mechanism of interaction between midday napping and hypertension deserves more attention as does investigating of other implications of midday napping considering its high prevalence.

## Appendix

Multimedia Appendix 1 Participant characteristics.

## Abbreviations

ADL: activities of daily living
CESD-10: Center for Epidemiological Studies Depression Scale
OR: odds ratio
