# Supplementary material for: The Association of Midday Napping With Hypertension Among Chinese Adults Older Than 45 Years: Cross-sectional Study
Source: JMIR Public Health Surveill. 2022 Nov 22;8(11):e38782. doi: 10.2196/38782 (PMC9727692; doi:10.2196/38782)
Supplement: Multimedia Appendix 1 [file publichealth_v8i11e38782_app1.docx]

**Multimedia Appendix 1.** Participant characteristics.

|  |  | 2011 (N) | | | | 2015 (N) | | | | | 2018 (N) | | | | |
| --- | --- | --- | --- | --- | --- | --- | --- | --- | --- | --- | --- | --- | --- | --- | --- |
|  |  | Total | No hypertension | Hypertension | P^a^ | Toal | No hypertension | Hypertension | P^a^ | Total | | No hypertension | Hypertension | P^a^ |  |
|  |  | N=11439 | N=8621 | N=2818 |  | N=12689 | N=10006 | N=2683 |  | N=9464 | | N=7372 | N=2092 |  |  |
| Gender |  |  |  |  | 0.556 |  |  |  | 0.851 |  | |  |  | 0.015 |  |
|  | Male | 5421 | 4072 | 1349 |  | 6265 | 4936 | 1329 |  | 4493 | | 3451 | 1042 |  |  |
|  | Female | 6018 | 4549 | 1469 |  | 6424 | 5070 | 1354 |  | 4971 | | 3921 | 1050 |  |  |
| Age (mean, years) |  | 59.53 | 59.56 | 59.45 | 0.600 | 61.00 | 61.02 | 60.95 | 0.738 | 60.58 | | 59,88 | 63,03 | p<0.001 |  |
| Education |  |  |  |  | 0.070 |  |  |  | 0.867 |  | |  |  | 0.311 |  |
|  | Never attended school | 3225 | 2475 | 750 |  | 3297 | 2615 | 682 |  | 1744 | | 1347 | 397 |  |  |
|  | Primary school and lower | 4653 | 3465 | 1188 |  | 4952 | 3889 | 1063 |  | 4281 | | 3330 | 951 |  |  |
|  | Junior middle school and lower | 2314 | 1726 | 588 |  | 1579 | 1245 | 334 |  | 2296 | | 1818 | 478 |  |  |
|  | Senior middle school and lower | 1247 | 955 | 292 |  | 2861 | 2257 | 604 |  | 1143 | | 877 | 266 |  |  |
| Residential status |  |  |  |  | 0.449 |  |  |  | 0.808 |  | |  |  | p<0.001 |  |
|  | Rural | 9250 | 6985 | 2265 |  | 9411 | 7426 | 1985 |  | 7105 | | 5605 | 1500 |  |  |
|  | Urban | 2189 | 1636 | 553 |  | 3278 | 2580 | 698 |  | 2359 | | 1767 | 592 |  |  |
| Marital status |  |  |  | 0.046 |  |  |  |  | 0.277 |  | |  |  | p<0.001 |  |
|  | Married / Cohabiting | 9916 | 7509 | 2407 |  | 11026 | 8671 | 2355 |  | 8361 | | 6572 | 1789 |  |  |
|  | Divorced / Widowed / Separated | 1431 | 1041 | 390 |  | 1583 | 1269 | 314 |  | 1057 | | 766 | 291 |  |  |
|  | Never married | 92 | 71 | 21 |  | 80 | 66 | 14 |  | 46 | | 34 | 12 |  |  |
| Log(Income)  (mean,￥) |  | 3.73 | 3.74 | 3.73 | 0.403 | 3.76 | 3.76 | 3.76 | 0.848 | 3.86 | | 3,88 | 3,81 | p<0.001 |  |
| Health status |  |  |  |  | 0.448 |  |  |  | 0.926 |  | |  |  | p<0.001 |  |
|  | Very good | 350 | 271 | 79 |  | 789 | 629 | 160 |  | 1276 | | 1130 | 146 |  |  |
|  | Good | 1321 | 984 | 337 |  | 1382 | 1094 | 288 |  | 1239 | | 1067 | 172 |  |  |
|  | Fair | 3654 | 2731 | 923 |  | 3988 | 3153 | 835 |  | 4698 | | 3701 | 997 |  |  |
|  | Poor | 4188 | 3190 | 998 |  | 4534 | 3564 | 970 |  | 1745 | | 1150 | 595 |  |  |
|  | Very poor | 1926 | 1445 | 481 |  | 1996 | 1566 | 430 |  | 506 | | 324 | 182 |  |  |
| ADL impaired |  |  |  |  | 0.312 |  |  |  | 0.825 |  | |  |  | p<0.001 |  |
|  | Yes | 3182 | 2419 | 763 |  | 4229 | 3330 | 899 |  | 2616 | | 1811 | 805 |  |  |
|  | No | 8257 | 6202 | 2055 |  | 8460 | 6676 | 1784 |  | 6848 | | 5561 | 1287 |  |  |
| Mental health |  |  |  |  | 0.611 |  |  |  | 0.081 |  | |  |  | p<0.001 |  |
|  | No depressive symptoms | 6720 | 5053 | 1667 |  | 7784 | 6099 | 1685 |  | 5950 | | 4766 | 1184 |  |  |
|  | Depressive symptoms | 4719 | 3568 | 1151 |  | 4905 | 3907 | 998 |  | 3514 | | 2606 | 908 |  |  |
| Diabetes |  |  |  |  | 0.535 |  |  |  | 0.006 |  | |  |  | p<0.001 |  |
|  | Yes | 659 | 490 | 169 |  | 745 | 558 | 187 |  | 1184 | | 592 | 592 |  |  |
|  | No | 10780 | 8131 | 2649 |  | 11944 | 9448 | 2496 |  | 8280 | | 6780 | 1500 |  |  |
| CVD |  |  |  |  | 0.156 |  |  |  | 0.004 |  | |  |  | p<0.001 |  |
|  | Yes | 1553 | 1148 | 405 |  | 1718 | 1310 | 408 |  | 2053 | | 1158 | 895 |  |  |
|  | No | 9886 | 7473 | 2413 |  | 10971 | 8696 | 2275 |  | 7411 | | 6214 | 1197 |  |  |
| BMI |  |  |  |  | 0.090 |  |  |  | p<0.001 |  | |  |  | p<0.001 |  |
|  | Underweight | 811 | 596 | 215 |  | 752 | 583 | 169 |  | 485 | | 430 | 55 |  |  |
|  | Normal weight | 7125 | 5385 | 1740 |  | 7476 | 6016 | 1460 |  | 5712 | | 4787 | 925 |  |  |
|  | Overweight ‎ | 2941 | 2196 | 745 |  | 3785 | 2905 | 880 |  | 2795 | | 1915 | 880 |  |  |
|  | Obese | 562 | 444 | 118 |  | 676 | 502 | 174 |  | 472 | | 240 | 232 |  |  |
| Drink |  |  |  |  | p<0.001 |  |  |  | p<0.001 |  | |  |  | 0.115 |  |
|  | Drink more than once a month | 2762 | 2153 | 609 |  | 3406 | 2774 | 632 |  | 2581 | | 2040 | 541 |  |  |
|  | Drink but less than once a month | 890 | 710 | 180 |  | 1121 | 949 | 172 |  | 753 | | 597 | 156 |  |  |
|  | Non-drinker | 7787 | 5758 | 2029 |  | 8162 | 6283 | 1879 |  | 6130 | | 4735 | 1395 |  |  |
| Smoke |  |  |  |  | 0.605 |  |  |  | 0.700 |  | |  |  | 0.002 |  |
|  | Yes | 4572 | 3434 | 1138 |  | 5646 | 4461 | 1185 |  | 4048 | | 3092 | 956 |  |  |
|  | No | 6867 | 5187 | 1680 |  | 7043 | 5545 | 1498 |  | 5416 | | 4280 | 1136 |  |  |
| Night sleep duration |  |  |  |  | 0.586 |  |  |  | 0.743 |  | |  |  | 0.001 |  |
|  | <6 | 3335 | 2528 | 807 |  | 3887 | 3065 | 822 |  | 3148 | | 2376 | 772 |  |  |
|  | 6-7 | 2356 | 1795 | 561 |  | 2620 | 2061 | 559 |  | 2185 | | 1723 | 462 |  |  |
|  | 7-8 | 2207 | 1662 | 545 |  | 2250 | 1770 | 480 |  | 1661 | | 1316 | 345 |  |  |
|  | 8-9 | 2393 | 1781 | 612 |  | 2593 | 2035 | 558 |  | 1736 | | 1390 | 346 |  |  |
|  | >9 | 1148 | 855 | 293 |  | 1339 | 1075 | 264 |  | 734 | | 567 | 167 |  |  |
| Midday napping duration |  |  |  |  | p<0.001 |  |  |  | p<0.001 |  | |  |  | p<0.001 |  |
|  | 0 | 5213 | 3906 | 1307 |  | 5280 | 4293 | 987 |  | 3737 | | 3011 | 726 |  |  |
|  | <30 | 1039 | 777 | 262 |  | 825 | 634 | 191 |  | 801 | | 610 | 191 |  |  |
|  | 30-60 | 994 | 762 | 232 |  | 1396 | 1075 | 321 |  | 1027 | | 794 | 233 |  |  |
|  | 60-90 | 2717 | 2101 | 676 |  | 3480 | 2697 | 783 |  | 2236 | | 1714 | 522 |  |  |
|  | >90‎ | 1416 | 1073 | 343 |  | 1708 | 1307 | 401 |  | 1663 | | 1243 | 420 |  |  |

N.B. ^a^: outcomes of Chi-square test and independent one sample t-test.
